# Effects of BCL-2 and MCL-1 Inhibition on Apoptotic and Transcriptional Profiles in Acute Myeloid Leukemia

**DOI:** 10.3390/medicina62071425

**Published:** 2026-07-22

**Authors:** Giedrė Skliutė, Eigintė Kuklytė, Andrius Žučenka, Veronika Viktorija Borutinskaitė, Rūta Navakauskienė

**Affiliations:** 1 Department of Molecular Cell Biology, Institute of Biochemistry, Life Sciences Center, Vilnius University, Saulėtekio Av. 7, LT-10257 Vilnius, Lithuania; 2 Department of Hematology and Oncology, Institute of Clinical Medicine, Faculty of Medicine, Vilnius University, LT-03101 Vilnius, Lithuania

**Keywords:** acute myeloid leukemia, BCL-2, MCL-1, apoptosis, cancer

## Abstract

*Background and Objectives*: Acute myeloid leukemia (AML) is characterized not only by its heterogeneity but also by its high relapse rate. This results in limited treatment options, especially in elderly or therapy-refractory patients. It is known that inhibiting anti-apoptotic BCL-2 family proteins can be effective; however, cellular resistance mechanisms often limit the efficacy of this treatment. We studied the effects of the BCL-2 inhibitor ABT-737, the MCL-1 inhibitor S63845, and their combination on AML cell lines and primary AML patient cells. *Materials and Methods*: To analyze the effects of ABT-737 and S63845 treatment on cells, cell energy phenotype, apoptosis, and cell cycle were assessed, and gene expression by RT-qPCR and protein levels by Western blot analysis were measured. *Results*: Treatment with the BCL-2 inhibitor ABT-737, the MCL-1 inhibitor S63845, and their combination reduced AML cell viability and induced apoptosis. Dual treatment also altered the expression of epigenetic regulators, as the levels of DNMT1, EZH2, SUZ12, and HDAC1 were reduced, while histone acetylation was increased. An increase in pro-apoptotic markers (PARP cleavage, caspase-9) was observed, and the expression of oncogenes (*MYC*, *WT1*) was reduced in model cell lines and primary AML patient cells. *Conclusions*: BCL-2 and MCL-1 inhibition, alone or in combination, induced apoptosis and altered the expression of epigenetic regulators and oncogenes in AML cell lines and primary patient cells, with no consistent advantage of combined treatment over single agents. BCL-2/MCL-1 inhibition remains a promising approach for AML, and further work should clarify which patients or disease subtypes are most likely to benefit from combined versus single-agent treatment.

## 1. Introduction

Acute myeloid leukemia (AML) is a complex disease characterized by uncontrolled proliferation of white blood cell precursors, especially in the bone marrow [1]. Despite advances in treatment, the prognosis of AML remains poor, especially in older patients who are often unable to receive intensive chemotherapy [2,3]. Recently, targeted therapies such as FLT3, IDH1/2 or BCL-2 inhibitors and novel immunotherapeutic strategies have been used [4,5,6,7,8,9,10,11]. However, therapeutic resistance remains a common problem, and approximately 50% of patients remain without adequate treatment [12]. Therefore, searching for new methods that would more effectively eliminate leukemic cells while preserving normal hematopoiesis is necessary.

Anti-apoptotic BCL-2 family proteins, including BCL-2, BCL-xL, BCL-w and MCL-1, are frequently overexpressed in leukemic cells and contribute to disease progression and resistance to treatment [13]. Inhibition of BCL-2 by the small molecule venetoclax has already shown clinical benefit, particularly in combination with hypomethylating agents [14]. However, resistance to venetoclax often develops due to compensatory overexpression of MCL-1 [13]. For this reason, selective MCL-1 inhibitors, such as S63845, have been developed and show strong preclinical potential [15].

Although dual inhibition of BCL-2 and MCL-1 has been proposed as a strategy [16] to overcome resistance, its broader impact on the molecular mechanisms of leukemia—particularly at the level of epigenetic regulation—remains poorly understood. Epigenetic reprogramming is a key feature of AML pathogenesis and therapeutic response; therefore, determining the relationship between BCL-2/MCL-1 inhibition and these processes could provide new insights into the feasibility of new antileukemic therapeutic strategies [17,18].

In this study, we investigated the effects of the BH3 mimetic (BCL-2/BCL-xL/BCL-w inhibitor) ABT-737 [19] and the MCL-1 inhibitor S63845 [20], individually and in combination, in AML model systems. We assessed cell viability, apoptosis, metabolism, and cell cycle progression in NB4, KG1, and chemotherapy-resistant KG1A cell lines, as well as in primary AML cells from patients. We also analyzed changes in the expression of genes and proteins involved in apoptosis, cell cycle, and epigenetic regulation. We aimed to determine whether dual inhibition of BCL-2 and MCL-1 not only enhances apoptosis but is linked to alterations in epigenetic regulatory pathways, providing further insight into the molecular responses associated with BCL-2 and MCL-1 inhibition in AML.

## 2. Materials and Methods

### 2.1. AML Cell Cultivation and Treatment with Agents

NB4 cells were cultivated in RPMI-1640 medium (ATCC, Manassas, VA, USA) with 10% FBS, KG1 and KG1A cells in IMDM medium (ATCC, Manassas, VA, USA) with 20% FBS, 100 μg/mL streptomycin and 100 U/mL penicillin (Gibco, Waltham, MA, USA) were added to both culture mediums. AML patients’ mononuclear cells were purified from blood or bone marrow using Ficoll-Paque PLUS density gradient centrifugation (GE Healthcare, Chicago, IL, USA). Primary AML mononuclear cells were processed immediately after Ficoll isolation (same day) and then allowed to recover overnight (≤16 h) prior to initiating drug exposure (defined as time 0). The study was conducted following the Declaration of Helsinki, and the protocol was approved by the Vilnius Regional Biomedical Research Ethics Committee (Approval No. 158200-16-824-356). Informed consent was obtained from all individual participants included in the study. Patients’ cells were cultivated in RPMI-1640 medium (ATCC, Manassas, VA, USA) with 10% FBS, 100 μg/mL streptomycin, and 100 U/mL penicillin (Gibco, Waltham, MA, USA). All cells were incubated at 37 °C, with 95% humidity and 5% CO_2_ atmosphere. For ABT-737 (Selleck Chemicals, Berlin, Germany) and S63845 (Cayman Chemical Company, Ann Arbor, MI, USA) treatment, cells were seeded at a 0.5 × 10^6^ cells/mL density. Cell count and viability were evaluated by mixing 40 μL of cell suspension with 40 μL of 0.2% trypan blue (Pharmacia LKB, Uppsala, Sweden) and manually counting unstained (viable) and stained (nonviable) cells with a hemocytometer. The effects of exposure to the researched agents were evaluated every 24 h for 72 h. NB4 cells were treated with 200 nM ABT-737, 20 nM S63845, or a combination of 20 nM ABT-737 and 2 nM S63845. KG1 cells were treated with 200 nM ABT-737, 200 nM S63845, or a combination of 20 nM ABT-737 and 20 nM S63845. KG1A cells were treated with 200 nM ABT-737, 1500 nM S63845 or a combination of 20 nM ABT-737 and 150 nM S63845. Patients’ cells were treated with 200 nM ABT-737, 200 nM S63845, or a combination of 20 nM ABT-737 and 20 nM S63845. Drug doses were chosen based on prior dose-finding experiments performed in our laboratory (for more information, refer to Supplementary Figure S1), aiming to approximate the IC_50_ (or sub-IC_50_) range for each cell line and to ensure: (i) a measurable biological response over 24–72 h, and (ii) sufficient viable cell numbers for downstream readouts. For combination conditions we intentionally used lower, sub-IC_50_-range doses (relative to single-agent doses) to allow detection of interaction effects while maintaining viable cells for mechanistic assays.

### 2.2. Cell Energy Phenotype Measurement

Cell extracellular acidification and oxygen consumption were measured using Agilent Seahorse XF Cell Energy Phenotype Test kit (Agilent Technologies, Santa Clara, CA, USA) according to the manufacturer’s instructions. Briefly: 24 h before the measurement, poly-D-lysin-coated plates were prepared. AML cell lines were treated with apoptosis-inducing agents and harvested after 24 and 72 h of treatment. Cells were seeded at 3.5 × 10^5^ cells/well, centrifuged (300× *g*, 1 min, RT) and incubated for 1 h at 37 °C without CO_2_. After incubation, the cells’ metabolism was evaluated using Agilent Seahorse XFp analyser (Agilent Technologies, Santa Clara, CA, USA).

### 2.3. Apoptosis and Cell Cycle Evaluation

Apoptosis was detected using the ApoFlowEx FITC kit (Exbio, Prague, Czech Republic) according to the manufacturer’s instructions. Stained cells were analyzed with a Guava EasyCyte (Millipore, Darmstadt, Germany) flow cytometer with Flowing Software 2 (Turku Bioscience 2018^©^, Turku, Finland). For cell cycle analysis, 2 × 10^6^ cells were centrifuged (300× *g*, 5 min, 4 °C), washed twice with PBS and resuspended in 1 mL of cold PBS. 3.3 mL of cold ethanol (96%) was slowly pipetted onto the cells and left to fixate overnight at −20 °C. After fixation, cells were washed twice (200 × g, 10 min, 4 °C) and resuspended in 500 μL PBS with PI (50 μg/mL) and RNase A (50 μg/mL). After 30 min of incubation at 37 °C, cells were analyzed with a Guava EasyCyte (Millipore, Darmstadt, Germany) flow cytometer.

### 2.4. Analysis of Gene Expression by RT-qPCR

Total cellular RNA was isolated using the Trizol reagent (Invitrogen, Waltham, MA, USA). Total RNA was treated with “DNase I, RNase-free (1 U/μL)” (Thermo Scientific, Waltham, MA, USA) kit to remove any traces of genomic DNA. Later, cDNA was synthesized using “LunaScript^®^ RT SuperMix” kit (New England Biolabs, Ipswich, MA, USA) and qPCR was performed using “Luna^®^ Universal qPCR Master Mix” kit (New England Biolabs, Ipswich, MA, USA) on the RotorGene 6000 system (Corbett Life Science, QIAGEN, Hilden, Germany). mRNA levels were normalized to *GAPDH* expression, and relative gene expression was calculated by the ∆∆Ct method. The primer sequences (Metabion International AG, Planegg, Germany) are listed in Supplementary Table S1.

### 2.5. Western Blot Analysis

To prepare the cell lysate, 2 × 10^6^ cells were centrifuged (350× *g*, 5 min), washed three times with cold PBS. 10 μL of supernatant was resuspended with 1 μL of benzonase (Merck, Darmstadt, Germany) and incubated for 30 min on ice. After incubation, 11 μL of 2X SDS lysis buffer and 178 μL of 1X lysis buffer were added, and the lysate was homogenized with an insulin syringe. After homogenization, the cell lysate was incubated (95 °C, 5 min) and centrifuged (20,000× *g*, 5 min). Proteins were fractionated on a 7–15% gradient polyacrylamide SDS-PAGE gel using Tris-glycine buffer. Afterwards, the electrophoresis was run, proteins were transferred to a PVDF membrane (Millipore, Burlington, MA, USA), which was blocked with 4% milk (Sigma-Aldrich, St. Louis, MO, USA) in PBS 0.05% Tween-20 and incubated with primary antibody. Later, the membrane was washed three times in PBS with 0.1% Tween-20 (AMRESCO, Solon, OH, USA) and then incubated with the secondary antibody. The list of antibodies is presented in Supplementary Table S2. Membrane was washed three times in PBS 0.1% Tween and once in PBS. Protein bands were detected using the protein detection kit WesternBrightTM ECL (Advansta, San Jose, CA, USA). Bands were imaged and intensity was calculated using ImageLab software v.6.1 (Bio-Rad, Hercules, CA, USA).

### 2.6. Statistical Analysis

Data are expressed as mean ± standard deviation. One-way ANOVA and Two-way ANOVA followed by Dunnett’s multiple comparisons test were used to calculate the significance of the difference between treated and control samples; significance was set at *p* ≤ 0.05 (*), *p* ≤ 0.01 (**), *p* ≤ 0.001 (***), *p* ≤ 0.0001 (****).

## 3. Results

In this study, we aimed to compare the effects of individual and combined BCL-2 and MCL-1 inhibition in AML. All of this was studied in different AML cell line models, including NB4: acute promyelocytic leukemia cells, that carry the t(15;17) PML-RARα fusion gene; KG1 acute myeloid leukemia cells, that are ATRA unresponsive, express fusion gene *FGFR1OP2-FGFR1*, *DNMT3A* and *KMT2A*; and chemotherapy-resistant, less differentiated cells that are unresponsive to CSF and do not express HLA—KG1A [21]. Primary cells from AML patients were also tested. To approximate the IC_50_ value for each line, cells were treated with ABT-737, S63845, or a combination of both agents: for NB4 cells—200 nM ABT-737, 20 nM S63845 or 20 nM ABT-737 + 2 nM S63845; for KG1 cells—200 nM ABT-737, 200 nM S63845 or 20 nM ABT-737 + 20 nM S63845; for KG1A cells, known for their greater resistance—200 nM ABT-737, 1500 nM S63845 or 20 nM ABT-737 + 150 nM S63845. We subsequently monitored changes in cell count, viability, apoptosis, and metabolic activity, and extended the analysis to gene and protein expression. Particular attention was paid to apoptosis signaling and transcriptional changes in apoptosis-related genes, epigenetic regulators, and oncogenes to determine whether BCL-2 and MCL-1 inhibition is associated with broader molecular responses in leukemic cells.

### 3.1. Effects of BCL-2 and MCL-1 Inhibition on AML Cell Survival, Apoptosis, and Cell Cycle

**Figure 1:**
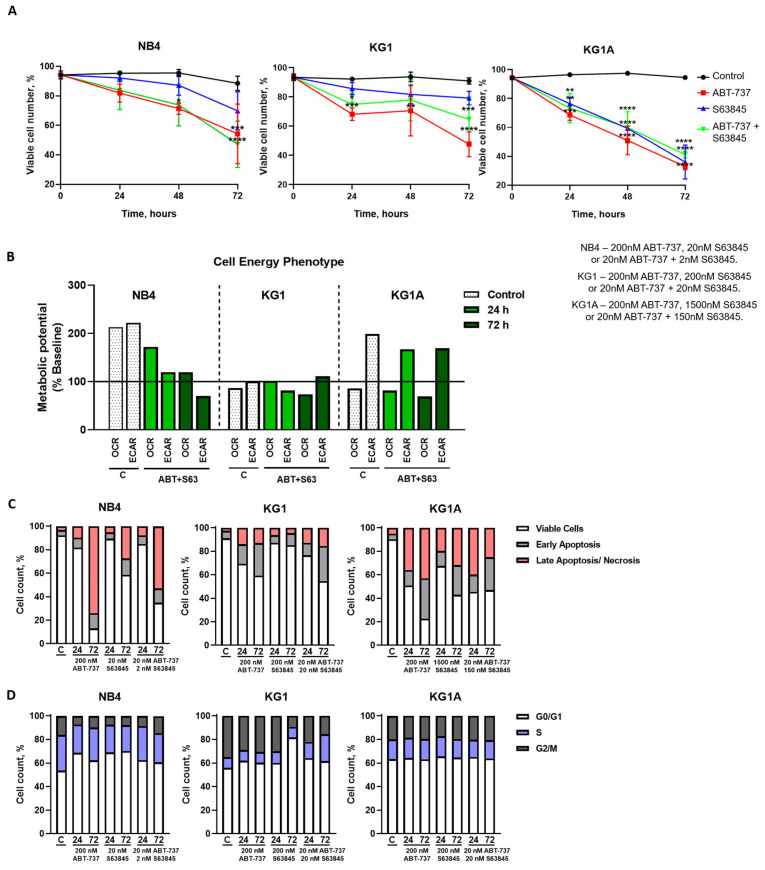
Cell viability, metabolic activity, apoptosis and cell cycle distribution of AML cell lines (NB4, KG1, KG1A) after treatment with ABT-737, S63845, or their combination. (**A**) Viability of NB4, KG1, and KG1A AML cell lines after treatment with ABT-737 (ABT), S63845 (S63), or their combination for 72 h (*n* = 3). Control (C)—untreated cells. Cells were treated with ABT-737 (ABT), S63845 (S63), or their combination for up to 72 h. NB4 cells—200 nM ABT-737, 20 nM S63845 or 20 nM ABT-737 + 2 nM S63845; KG1 cells—200 nM ABT-737, 200 nM S63845 or 20 nM ABT-737 + 20 nM S63845; KG1A cells 200 nM ABT-737, 1500 nM S63845 or 20 nM ABT-737 + 150 nM S63845. Statistical significance was determined using two-way ANOVA followed by Dunnett’s multiple comparisons test, *p* ≤ 0.05 (*), *p* ≤ 0.01(**), *p* ≤ 0.001 (***), *p* ≤ 0.0001 (****). (**B**) Metabolic activity of AML cells assessed by oxygen consumption rate (OCR) and extracellular acidification rate (ECAR) after 24 h and 72 h of treatment. Data are presented as the mean (*n* = 2). (**C**) Apoptosis analysis of NB4, KG1, and KG1A cells treated with ABT-737, S63845, or their combination for 24 h or 72 h. Annexin V–FITC and propidium iodide staining were used to distinguish viable, early, and late apoptotic/necrotic cells, expressed as percentages of the total population. (**D**) Cell cycle distribution of NB4, KG1, and KG1A cells after 24 h or 72 h of treatment with ABT-737, S63845, or their combination. Cells were analyzed by propidium iodide DNA staining and classified into G0/G1, S, and G2/M phases. Control (C) represents untreated cells. Results are presented as mean values from three independent experiments.

To evaluate the effect of BCL-2 and MCL-1 inhibition on AML cell survival, we first analyzed cell viability over a 72 h treatment period. Treatment with ABT-737 (ABT), S63845 (S63), or their combination resulted in a progressive decrease in viable cells across all three cell lines tested. In KG1 cells, the greatest reduction in viability was observed with ABT-737 alone, which reduced viability to approximately half the level of control cells after 72 h. At the same time, the combination with S63845 showed a slightly lower effect. A similar response was observed in NB4 cells, with viability decreasing to approximately 50% after prolonged exposure, whether with ABT-737 alone or in combination. In contrast, chemotherapy-resistant KG1A cells required higher drug concentrations to achieve a marked reduction in viability, which decreased to approximately one-third of control levels after 72 h of treatment (Figure 1A).

Cell number variation was also assessed (Supplementary Figure S2). ABT-737 and S63845 lowered the cell count of NB4 and KG1 cells, whereas KG1A cells required higher concentrations to achieve a similar effect. These results were consistent with the observed decrease in viability and indicate that BCL-2 and MCL-1 inhibition effectively inhibits AML cell growth.

We next investigated whether the BCL-2/MCL-1 inhibition affected cellular metabolism by measuring oxygen consumption rate (OCR; mitochondrial respiration) and extracellular acidification rate (ECAR; glycolysis). In KG1 and KG1A cells, metabolic activity remained largely unchanged, with OCR and ECAR values similar to those of untreated control cells. In contrast, NB4 cells showed changes in metabolic activity: ECAR values decreased after 24 h of combined ABT-737 and S63845 treatment, while a reduction in OCR was observed after 72 h. These findings suggest that combined BCL-2/MCL-1 inhibition may reduce both glycolytic and mitochondrial respiratory activity in NB4 cells (Figure 1B).

We further investigated whether ABT-737, S63845, and their combination induce apoptosis in leukemic cells by flow cytometry using annexin V–FITC and propidium iodide staining. In KG1 cells, 72 h of treatment with ABT-737 or ABT-737 + S63845 increased the proportion of cells in the early stage of apoptosis to almost 30%, whereas S63845 alone had little effect (Figure 1C). In KG1A cells, treatment with ABT-737, S63845, or their combination increased the proportion of apoptotic and late apoptotic/necrotic cells after both 24 and 72 h, with the greatest effect observed following ABT-737 treatment (Figure 1C). In NB4 cells, the most pronounced response was observed after 72 h of treatment with the ABT-737 inhibitor—the proportion of cells in the late apoptosis/necrosis stage reached about 70%. S63845 or ABT-737 + S63845 treatment also increased the proportion of late apoptosis, albeit to a lesser extent (Figure 1C).

Cell cycle analysis showed that all treatments had minimal effects on the distribution of cells in different cell cycle fractions (Figure 1D). In NB4 cells, S63845 treatment increased the proportion of cells in the G0/G1 phase compared with the control. A similar trend was observed in KG1 cells, where at 72 h S63845 treatment increased the proportion in the G0/G1 phase by about 30% compared to the control. In contrast, no substantial changes were observed in KG1A cells—about 60% of cells remained in the G0/G1 phase after treatment with the inhibitors (Figure 1D).

In conclusion, it can be stated that BCL-2 and MCL-1 inhibition reduces the viability of AML cells and, in the case of NB4 cells, inhibits metabolic activity. Apoptosis induction was clearly observed in all three model systems, but the most pronounced effect was observed in chemotherapy-resistant KG1A cells and NB4 cells after prolonged exposure to ABT-737. Meanwhile, the impact on cell cycle distribution was only minor and cell line dependent. Our results suggest that inhibition of BCL-2 and MCL-1 primarily acts through apoptosis induction rather than through significant changes in cell cycle progression.

### 3.2. Gene Expression Changes Induced by BCL-2 and MCL-1 Inhibition

Next, we investigated transcriptional changes associated with the observed reduction in cell proliferation and viability. We analyzed the expression of selected genes involved in key regulatory pathways. Our focus was on three groups of genes: (a) apoptosis-related genes (*BAX*, *BCL-2*, *BCL-xL*, *MCL-1* and *APAF1*), (b) epigenetic regulators (*SIN3A*, *GATAD2A*, *TET1*, *KAT6A*, *HDAC2*), and (c) cell cycle regulators and oncogenes (*TP53*, *CDKN1A*, *IDH1*, *MYC*, *WT1*). This approach allowed us to assess whether BCL-2 and MCL-1 inhibition was associated with transcriptional changes in apoptosis-related genes, epigenetic regulators, and genes involved in leukemic cell fate.

**Figure 2:**
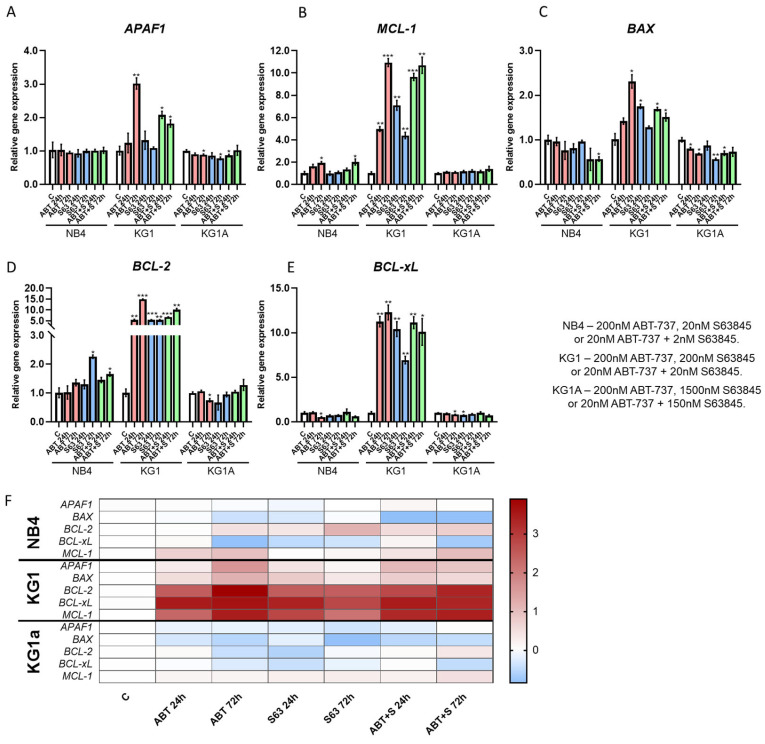
Apoptosis-related gene expression in AML cell lines following treatment with ABT-737 and S63845. (**A**–**E**) RT-qPCR of *BAX*, *BCL-2*, *BCL-xL*, *MCL-1*, and *APAF1* in NB4, KG1, and KG1A cells after 24 h and 72 h of treatment with ABT-737 (ABT), S63845 (S), or the combination (ABT+S). Control (C)—untreated cells. NB4 cells—200 nM ABT-737, 20 nM S63845 or 20 nM ABT-737 + 2 nM S63845; KG1 cells—200 nM ABT-737, 200 nM S63845 or 20 nM ABT-737 + 20 nM S63845; KG1A cells 200 nM ABT-737, 1500 nM S63845 or 20 nM ABT-737 + 150 nM S63845. Data are mean ± S.D. (*n* = 3). Statistical significance was assessed by one-way ANOVA followed by Dunnett’s multiple comparisons test, *p* ≤ 0.05 (*), *p* ≤ 0.01 (**), *p* ≤ 0.001 (***). (**F**) Summary heatmap showing log2(fold change) relative to the matched control for the same genes across all cell lines and conditions. Colors indicate direction and magnitude of change (blue, down; white, no change; red, up). Columns are ordered C → ABT (24 h, 72 h) → S (24 h, 72 h) → ABT+S (24 h, 72 h); rows are grouped by cell line (KG1, KG1A, NB4) with genes listed per group. The heatmap is provided for pattern visualization; statistical testing is reported in panels (**A**–**E**).

To better understand the effects of BCL-2 and MCL-1 inhibition on apoptotic signaling, we examined the expression of five key apoptosis-related genes, *APAF1*, *BAX*, *BCL-2*, *BCL-xL* and *MCL-1* by RT-PCR (Figure 2A–E). In NB4 cells, the changes were moderate: after 72 h of treatment with S63845 or in combination with ABT-737, *BCL-2* and *MCL-1* expression increased, while *BCL-xL* and *BAX* levels tended to decrease slightly. Treated with ABT-737 alone or in combination with S63845, KG1 cells displayed the strongest transcriptional response, characterized by increased expression of both pro-apoptotic (*APAF1*, *BAX*) and anti-apoptotic (*BCL-2*, *BCL-xL*, *MCL-1*) genes. These findings may reflect a compensatory transcriptional response to apoptosis induction. In contrast, KG1A cells showed only minimal changes: *BAX* expression tended to decrease, while *BCL-2*, *BCL-xL*, *MCL-1*, and *APAF1* gene expression levels remained mostly stable. Heatmap visualization highlighted the marked increase in *APAF1*, *BCL-2*, *BCL-xL*, and *MCL-1* induction in KG1 cells, in contrast to the relatively stable profiles of NB4 and KG1A. In conclusion, treatment with BCL-2 and MCL-1 inhibitors resulted in the most pronounced transcriptional changes in KG1 cells, whereas transcriptional changes were comparatively limited in KG1A cells.

**Figure 3:**
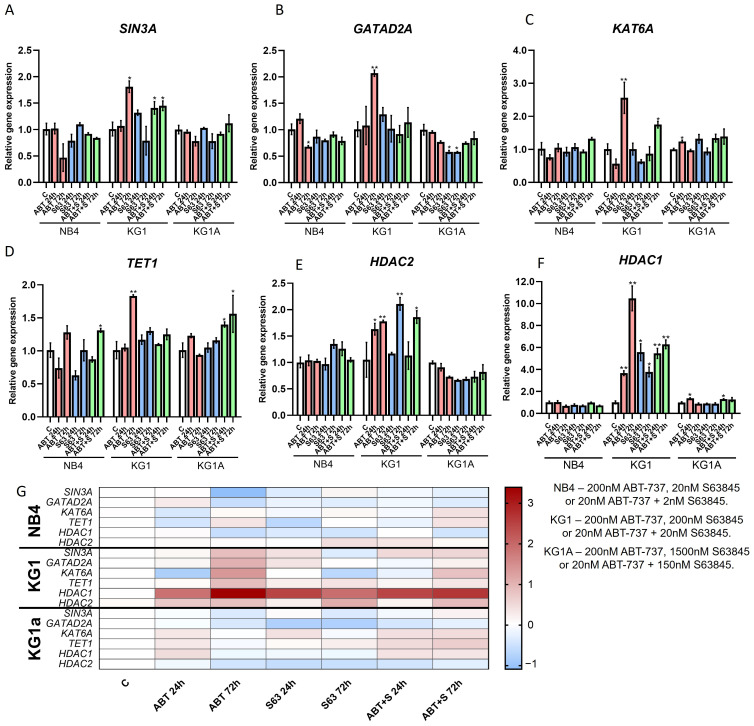
Expression of genes involved in epigenetic regulation after treatment with ABT-737, S63845, or their combination. (**A**–**F**) Real-time PCR analysis of *SIN3A*, *GATAD2A*, *TET1*, *KAT6A*, *HDAC1* and *HDAC2* expression in NB4, KG1, and KG1A AML cell lines following treatment for 24 h or 72 h. Results are shown as relative expression compared to untreated controls (C). ABT—ABT-737; S—S63845; ABT+S—combination of agents. Data represent mean ± S.D. (*n* = 3). NB4 cells—200 nM ABT-737, 20 nM S63845 or 20 nM ABT-737 + 2 nM S63845; KG1 cells—200 nM ABT-737, 200 nM S63845 or 20 nM ABT-737 + 20 nM S63845; KG1A cells 200 nM ABT-737, 1500 nM S63845 or 20 nM ABT-737 + 150 nM S63845. Statistical significance was determined using one-way ANOVA followed by Dunnett’s multiple comparisons test, *p* ≤ 0.05 (*), *p* ≤ 0.01 (**). (**G**) Summary heatmap showing log2(fold change) relative to the matched control for the same genes across all cell lines and conditions. Colors indicate direction and magnitude of change (blue, down; white, no change; red, up). Columns are ordered C → ABT (24 h, 72 h) → S (24 h, 72 h) → ABT+S (24 h, 72 h); rows are grouped by cell line (KG1, KG1A, NB4) with genes listed per group. The heatmap is provided for pattern visualization; statistical testing is reported in panels (**A**–**F**).

The effects of ABT-737 and S63845 inhibitors on regulating epigenetic processes were further investigated. We analyzed the changes in gene expression of six representative epigenetic regulators—*SIN3A*, *GATAD2A*, *TET1*, *KAT6A*, *HDAC1* and *HDAC2*. In KG1 cells, ABT-737 treatment induced an apparent increase in *SIN3A* gene expression after 72 h. A similar trend was observed with the combined treatment of ABT-737 + S63845 (Figure 3). A significant increase in *GATAD2A* expression was also recorded after 72 h of ABT-737 treatment. No significant changes in *GATAD2A* expression were observed following the combined treatment. *TET1* expression increased after ABT-737 treatment, whereas no significant changes were observed following the combined treatment in KG1 cells. Similarly, after 72 h, ABT-737 or ABT-737 + S63845 significantly increased the expression of *KAT6A*, *HDAC1*, and *HDAC2* genes, suggesting a broader effect of the inhibitors on chromatin remodeling and histone modification pathways. In KG1A cells, gene expression changes were less pronounced. After 24 h and 72 h of S63845 exposure, a slight decrease in *GATAD2A* gene expression level was observed, while *TET1* expression slightly increased after ABT-737 + S63845 at both early and late time points. The expression of other genes—*SIN3A*, *KAT6A*, and *HDAC2*—was essentially unchanged compared to untreated control cells (Figure 3). No significant changes in *SIN3A* and *TET1* expression were observed in NB4 cells following any treatment, whereas *KAT6A* and *HDAC2* expression remained stable across all conditions (Figure 3C–F).

Heatmap visualization emphasized the prominent changes in epigenetic regulators in KG1 cells, particularly the increase in *TET1*, *KAT6A*, *HDAC1*, and *HDAC2* expression, compared to minimal or inconsistent changes in NB4 and KG1A cells (Figure 3G). These findings suggest that KG1 cells are transcriptionally responsive to *BCL-2* and *MCL-1* inhibition. In contrast, transcriptional changes were comparatively limited in KG1A and NB4 cells.

**Figure 4:**
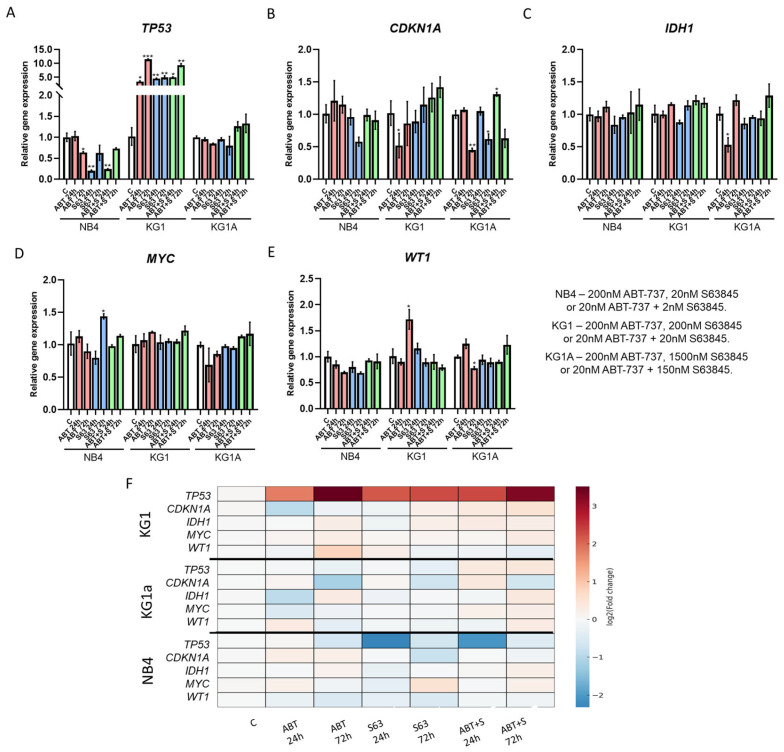
Expression of genes involved in cell cycle regulation and oncogenic signaling after treatment with ABT-737, S63845, or their combination. (**A**–**E**) Real-time PCR analysis of *TP53*, *CDKN1A*, *IDH1*, *MYC*, and *WT1* expression in NB4, KG1, and KG1A AML cell lines following treatment for 24 h or 72 h. Results are shown as relative expression compared to untreated controls (C). ABT—ABT-737; S—S63845; ABT+S—combination of agents. NB4 cells—200 nM ABT-737, 20 nM S63845 or 20 nM ABT-737 + 2 nM S63845; KG1 cells—200 nM ABT-737, 200 nM S63845 or 20 nM ABT-737 + 20 nM S63845; KG1A cells 200 nM ABT-737, 1500 nM S63845 or 20 nM ABT-737 + 150 nM S63845. Data represent mean ± S.D. (*n* = 3). Statistical significance was determined using one-way ANOVA followed by Dunnett’s multiple comparisons test, *p* ≤ 0.05 (*), *p* ≤ 0.01 (**), *p* ≤ 0.001 (***). (**F**) Summary heatmap showing log2(fold change) relative to the matched control for the same genes across all cell lines and conditions. Colors indicate direction and magnitude of change (blue, down; white, no change; red, up). Columns are ordered C → ABT (24 h, 72 h) → S (24 h, 72 h) → ABT+S (24 h, 72 h); rows are grouped by cell line (KG1, KG1A, NB4) with genes listed per group. The heatmap is provided for pattern visualization; statistical testing is reported in panels (**A**–**E**).

We next investigated how BCL-2 and MCL-1 inhibition affects transcriptional programs related to cell cycle control and oncogenic signaling: changes in *TP53*, *CDKN1A*, *IDH1*, *MYC*, and *WT1* gene expression in AML cell lines were analyzed (Figure 4A–E). We found that in KG1 cells, exposure to ABT-737 or the ABT-737 + S63845 combination increased *TP53* and *CDKN1A* expression, particularly after 72 h (Figure 4A,B). The effect was most pronounced after 72 h of treatment. *MYC* and *WT1* expression decreased under selected treatment conditions. In NB4 cells, gene expression changes were more moderate, but followed a similar pattern—a slight increase in *TP53/CDKN1A* and a decrease in *MYC* after combination treatment. KG1A cells showed the weakest transcriptional response. A reduction in WT1 expression was observed after prolonged treatment. These results demonstrate that BCL-2 and MCL-1 inhibition is associated with transcriptional changes in genes involved in cell cycle regulation and oncogenic signaling, with the most pronounced response observed in KG1 cells. Heatmap analysis further highlighted these differences, clearly visualizing the upregulation of *TP53* and *CDKN1A* and the downregulation of *MYC* and *WT1* in KG1 cells compared to relatively weaker changes in NB4 and KG1A (Figure 4F). Overall, transcriptional responses were more pronounced in KG1 cells than in NB4 or KG1A cells.

In summary, this section shows that treatment with BCL-2 and MCL-1 inhibitors is associated with transcriptional changes in apoptosis-related genes, epigenetic regulators, and genes involved in oncogenic signaling in AML cells. KG1A cells showed comparatively limited transcriptional changes, whereas NB4 displayed an intermediate response and KG1 exhibited the most pronounced changes, including increased *TP53*, *CDKN1A*, *APAF1*, and *BAX* expression together with reduced *MYC* and *WT1* expression. Together, these findings indicate that BCL-2 and MCL-1 inhibition is associated with coordinated transcriptional changes in genes involved in apoptosis, epigenetic regulation, and cell growth, with the most pronounced response observed in KG1 cells.

### 3.3. Protein-Level Validation of Apoptotic and Epigenetic Regulators Following BCL-2 and MCL-1 Inhibition

We further analyzed how the observed transcriptional changes are reflected at the protein level: NB4, KG1, and KG1A cells were analyzed for the expression of proteins related to apoptosis and epigenetic regulation after 24 and 72 h of treatment (Densitometric graphs in Supplementary Figures S3 and S4).

We found that the poly(ADP-ribose) polymerase cleavage product (cleaved PARP) was increased in NB4 and KG1 cells, most prominently after ABT-737 treatment. The level of cleaved caspase-9 was also increased in NB4 and KG1 cells, whereas this change was much weaker in chemotherapy-resistant KG1A cells. The level of BCL-2 protein in KG1 cells decreased after ABT-737 treatment, while it remained almost unchanged in NB4 and KG1A cells. Bax protein showed a slight decrease, most noticeably after ABT-737 or ABT-737 + S63845 treatment, whereas S63845 alone caused only minor changes. Taken together, the increased levels of cleaved PARP and caspase-9 in NB4 and KG1 cells are consistent with activation of apoptotic pathways. In contrast, although apoptosis was detected in KG1A cells by Annexin V staining, the absence of a marked increase in cleaved PARP and only minimal changes in cleaved caspase-9 suggest that apoptosis in this cell line may proceed through different molecular mechanisms or with different kinetics.

The importance of the Polycomb Repressive Complex 2 (PRC2) in AML progression is well established [22,23,24,25,26,27]. In addition, DNA methylation and histone deacetylation are key epigenetic mechanisms involved in leukemogenesis, in which DNMT1 [28,29] and HDAC1 [30,31] contribute to the maintenance of epigenetic regulation. In this study DNMT1 protein levels were consistently decreased in all AML lines, most significantly in NB4 cells after 72 h of ABT-737 or ABT-737 + S63845 exposure. In contrast, the KG1A line was most sensitive to S63845 exposure alone. EZH2 protein levels in NB4 cells decreased after ABT-737 and combination treatment, while SUZ12 levels decreased significantly in all lines after S63845 exposure. We found that HDAC1 protein expression in NB4 cells decreased more than 2-fold after 72 h of ABT-737 exposure, while changes were less pronounced in KG1 and KG1A cells. Accordingly, with the decrease in HDAC1, an increase in histone acetylation was detected in NB4 cells—the level of H4K16Ac increased. Meanwhile, H4 hyperacetylation was most pronounced in KG1 cells. No significant increase in acetylation was observed in KG1A cells (Densiometric graphs can be found in Supplementary Figures S3 and S4).

The protein analysis results complement some of the gene expression data and show that dual inhibition of BCL-2 and MCL-1 affects apoptosis pathways and epigenetic regulation. It was found that DNMT1, HDAC1, EZH2, and SUZ12 protein expression is reduced in NB4 and KG1 cells, and this decrease coincides with the RT-qPCR data. Also, with a reduction in HDAC1 levels in NB4 cells, an increase in histone acetylation (H4K16Ac) was observed, which probably corresponds to the initiation of more active transcriptional programs. In KG1 cells, where the most pronounced changes in gene expression were observed, the highest histone H4 hyperacetylation was also recorded, possibly indicating the connection of epigenetic rearrangement with the switching of transcriptional programs. However, the levels of BCL-2 protein and *BCL-2* gene were opposing—gene expression was significantly upregulated, whilst protein levels remained consistent or even downregulated after ABT-737 treatment. To observe if cells tend to lean towards apoptosis or survival, we calculated the BAX/BCL-2 ratio (Supplementary Figure S5). Data showed that KG1 cells treated with S63845 and the combination of ABT-737 and S63845 tend to lean towards apoptosis. Our results suggest that changes at the protein and histone modification levels are closely related to transcriptional responses and may contribute to the induction of apoptosis and to the sensitivity of leukemic cells to therapy.

### 3.4. Validation of BCL-2 and MCL-1 Inhibition Effects in Primary AML Patient Cells

We further extended our studies to primary leukemic cells from AML patients. Clinical data for the patient population are provided in Supplementary Table S3. This allowed us to assess whether the effects of BCL-2 and MCL-1 inhibitors observed in vitro are maintained ex vivo.

**Figure 5:**
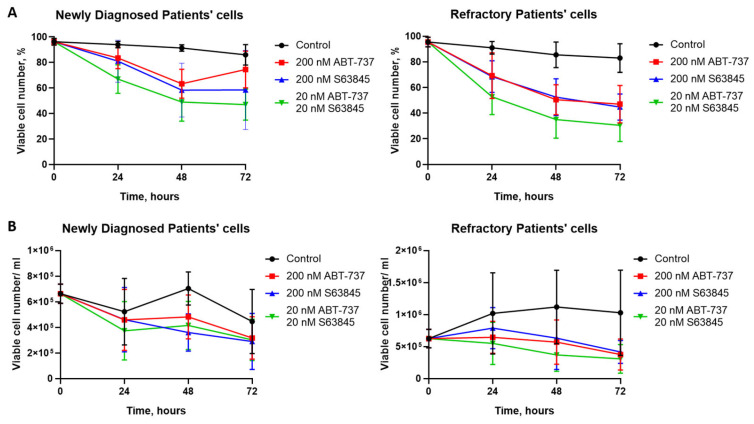
(**A**) Cell viability and (**B**) number of AML blasts from patients after treatment with ABT-737, S63845, or their combination. Blasts isolated from AML patients after treatment with ABT-737, S63845, or their combination for up to 72 h. Control (C)—untreated cells. Cells were treated with ABT-737, S63845, or their combination for up to 72 h. Data are presented as mean ± S.D. Differences between groups were analyzed by two-way ANOVA. (*n* = 4 for the newly diagnosed patients’ group, and *n* = 4 for the refractory patients’ group).

First, we treated cells with ABT-737, S63845, or their combination and assessed cell viability and number over 72 h. Treatment with ABT-737, S63845, or their combination reduced both cell viability and cell number compared with untreated controls. To determine whether disease status influenced treatment response, we compared blast samples from newly diagnosed and refractory AML patients. No statistically significant differences in viability or cell number were observed between these two groups under any treatment condition. However, it should be noted that blasts from refractory AML patients tend to be more sensitive to the treatments used, which may be related to the status-related influence of other treatments (Figure 5A,B).

**Figure 6:**
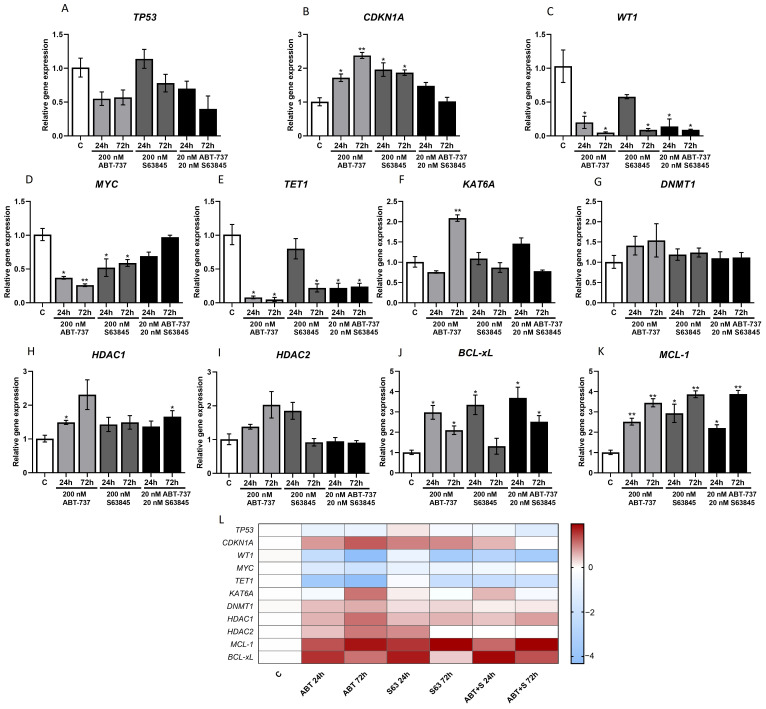
Expression of genes involved in cell cycle regulation and oncogenic signaling after treatment with ABT-737, S63845, or their combination. (**A**–**K**) Real-time PCR analysis of *TP53*, *CDKN1A*, *TET1*, *KAT6A*, *DNMT1*, *HDAC1*, *HDAC2*, *MYC*, *WT1*, *BCL-xL*, and *MCL-1* expression in AML patient cells following treatment for 24 h or 72 h. Results are shown as relative expression compared to untreated controls (C). ABT—ABT-737; S—S63845; ABT+S—combination of agents. Data represent mean ± S.D. (*n* = 3). Statistical significance was determined using one-way ANOVA followed by Dunnett’s multiple comparisons test, *p* ≤ 0.05 (*), *p* ≤ 0.01 (**). (**L**) Summary heatmap showing log2(fold change) relative to the matched control for the same genes across all cell lines and conditions. Colors indicate direction and magnitude of change (blue, down; white, no change; red, up). Columns are ordered C → ABT (24 h, 72 h) → S (24 h, 72 h) → ABT+S (24 h, 72 h); The heatmap is provided for pattern visualization; statistical testing is reported in panel (**A**–**K**).

We investigated the expression of genes *BCL-xL*, *MCL-1*, also genes involved in epigenetic processes (*TET1*, *KAT6A*, *HDAC2*, *HDAC1*, *DNMT1*), oncogenes (*MYC*, *WT1*), and cell cycle regulators (*TP53*, *CDKN1A*) (Figure 6). We showed that in AML patients cells expression of *BCL-xL* and *MCL-1* genes increased after treatment. *TP53* gene expression was reduced by almost half after ABT-737 or ABT-737 + S63845 treatment. At the same time, its downstream effector *CDKN1A* was significantly increased after ABT-737 or S63845 exposure, both after 24 and 72 h. The most pronounced change was in *WT1* expression—it was significantly reduced after 72 h of all exposures. *MYC* gene expression was also reduced after ABT-737 or S63845 treatment (24 and 72 h) but remained unchanged after the combined treatment. Among epigenetic regulators, *TET1* was decreased significantly after 72 h. after all treatments, an increase in *KAT6A* was observed only after ABT-737 (72 h), while *HDAC1* expression increased after ABT-737 treatment and 72 h of combined treatment. No significant changes were observed in *DNMT1* and *HDAC2*. A side-by-side heatmap (Figure 6L) summarizes these transcriptional patterns, highlighting the consistent downregulation of oncogenes and *TET1* and induction of *CDKN1A*. These data suggest that pro-apoptotic effects in AML patient cells reduce viability and reprogram transcriptional programs that regulate cell cycle and oncogenic signaling.

Together, these results demonstrate that patient-derived AML cells, like commercial AML model lines, are sensitive to dual inhibition of BCL-2 and MCL-1. Treatment reduced cell viability and proliferation, while simultaneously inducing transcriptional changes that included silencing oncogenes (*MYC*, *WT1*) and modulation of cell cycle regulators (*TP53*, *CDKN1A*). These data suggest that combined targeting of BCL-2 and MCL-1 may also confer therapeutic advantage in patient cells, opening the possibility of more efficient destruction of leukemic cells.

## 4. Discussion

Acute myeloid leukemia (AML) is a highly heterogeneous disease, which is associated with both diverse genetic alterations and individual response to treatment [1,2,3]. This heterogeneity complicates the development of appropriate therapies and highlights the importance of having clear models that accurately reflect the biology of patients’ cells [32]. Therefore, in this study, we selected three different AML cell lines—NB4 (APL subtype), KG1 (therapy-sensitive), and KG1A (chemoresistant)—and examined them alongside leukemic cells derived from AML patients. Our primary objective was to assess whether the response of patients’ cells could be reflected in cell lines and which of them most closely represent the biology of AML ex vivo.

BCL-2 family proteins are key regulators of apoptosis, maintaining a balance between pro-survival (BCL-2, BCL-xL, BCL-w, MCL-1, A1) and pro-apoptotic (BAX, BAK, BOK) members [18]. It has been shown that in leukemic cells, this balance is biased toward pro-survival, with overexpression of BCL-2 or MCL-1 often leading to inhibition of the activity of pro-apoptotic proteins [13]. As a result, these cells become resistant to natural death signals and gain an advantage in survival and proliferation, one of the leading causes of leukemogenesis and therapeutic resistance. BH3 mimetics have already shown therapeutic potential, but resistance often arises due to compensatory expression of other BCL-2 family members [33]. ABT-737 binds strongly to BCL-2, BCL-xL, and BCL-w, but not to MCL-1, while S63845 selectively acts on MCL-1 [17,34]. Therefore, their combination could theoretically affect multiple anti-apoptotic targets and reduce the risk of resistance [35]. Our results demonstrate that treatment with ABT-737, S63845, and their combination effectively reduced AML cell number and viability both in vitro and ex vivo, supporting the therapeutic potential of BCL-2/MCL-1 targeting.

At the functional level, inhibition of BCL-2 and MCL-1 induced signs of apoptosis in all tested cell lines, both AML and patient cells. We found increased levels of PARP and caspase-9 cleavage, most prominently in KG1 and patient AML cells. The KG1A line, corresponding to a chemoresistant phenotype, remained less sensitive. The response of patient cells was closer to KG1 than to NB4 or KG1A, suggesting that KG1 may be the most translationally relevant model among the commonly used AML cell lines. This is an important insight that helps to bridge the gap between preclinical models and patient biology. Although apoptosis was detected in KG1A cells by Annexin V staining, we did not observe a corresponding increase in cleaved PARP or cleaved caspase-9. This suggests that apoptosis in KG1A cells may involve molecular mechanisms distinct from those operating in NB4 and KG1 cells or may occur with different kinetics.

We also found that the expression of apoptosis genes (*BAX*, *APAF1*, *BCL-2*, *BCL-xL*, *MCL-1*) was affected differentially. In KG1 and patient cells, ABT-737 or ABT-737 + S63845 strongly induced *APAF1* and *CDKN1A* gene expression and increased *TP53* gene expression. That could indicate the activation of p53-dependent checkpoints. In contrast, KG1A cells remained largely unresponsive. An interesting finding was that in KG1 cells, *BCL-2* gene expression was increased but protein levels were decreased, suggesting possible posttranscriptional regulatory mechanisms and feedback effects [36]. This discrepancy could be explained by ubiquitin-dependent proteosome degradation of BCL-2 and protein turnover [37,38,39] or miRNA-mediated repression of BCL-2 expression [40,41,42]. More broadly, the transcriptional responses varied substantially among the three AML cell lines, whereas apoptosis was induced in all models. This suggests that the observed changes in epigenetic regulators and oncogenic signaling molecules are unlikely to represent a universal mechanism directly responsible for the induction of apoptosis. We believe it is more likely that these transcriptional shifts are cell line-specific, adaptive, or downstream responses to BCL-2/MCL-1 inhibition that accompany apoptosis rather than cause it. This fits with evidence that mitochondrial outer membrane permeabilization can be limited and sublethal, insufficient to kill the cell, but enough to drive downstream transcriptional and genomic changes [43], and that sublethal engagement of the apoptotic machinery can itself generate distinct transcriptional programs [44]. Additional studies are required to establish whether these molecular changes contribute functionally to treatment response or are secondary consequences of apoptosis induction.

The BAX/BCL-2 protein ratio differed among the three AML cell lines despite apoptosis being induced in all models. This suggests that alterations in the BAX/BCL-2 ratio are unlikely to represent a universal mechanism underlying apoptosis following BCL-2/MCL-1 inhibition. Instead, these changes may reflect cell line-specific molecular responses, whereas apoptosis is likely regulated by multiple pathways, depending on the biological characteristics of each AML model.

In addition to apoptosis, we also detected epigenetic changes. In KG1 cells, chromatin remodeling genes (*SIN3A*, *GATAD2A*, *TET1*, *KAT6A*, *HDAC2*, *HDAC1*) were consistently upregulated after ABT-737 or ABT-737 + S63845 exposure. At the same time, DNMT1, EZH2, and SUZ12 protein levels were downregulated in all lines, especially in NB4. These results are consistent with previous work showing that DNMT1 downregulation promotes p21/CDKN1A expression [45,46], EZH2 and SUZ12 (PRC2) promote H3K27me3-mediated repression of BIM, contributing to apoptosis resistance; conversely, their inhibition may restore BIM expression and apoptotic sensitivity [47,48], and *HDAC1* gene expression downregulation is associated with increased H4 protein acetylation [49]. Taken together, the epigenetic and transcriptional alterations that accompanied apoptosis induction in AML cells suggest an association rather than a direct causal relationship.

Observed molecular differences in treatment response across cell lines could be attributed to their properties, such as subtype, differentiation state, TP53 status, or baseline apoptotic dependencies. NB4 cells are APL FAB-M3, characterized by t(15;17)(q24;21) translocation and PML-RARα fusion protein, cells are at promyelocyte stage, but highly responsive to ATRA and have wild type TP53 signaling and highly apoptosis competent and responsive to BCL-2 inhibition [50,51,52]. KG1 cells are AML with minimal maturation FAB-M1, cells are at the early myeloblast stage, capable of limited differentiation, and altered TP53; they tend to rely on MCL-1 for apoptosis and are still sensitive to therapies [53,54]. Interestingly, in our study ABT-737 and S63845 caused more alterations in KG1 cells than in NB4 cells. KG1A cells are more immature than KG1 cells, resistant to differentiation, have altered TP53 signaling, and rely on MCL-1 and other survival signaling pathways; therefore, they generally respond poorly to apoptosis-inducing drugs [55,56,57]. In our study, however, KG1A cells required substantially higher concentrations of S63845 to achieve a pronounced reduction in viability and induction of apoptosis. This indicates that although KG1A cells are resistant to conventional chemotherapy, they remain susceptible to BCL-2/MCL-1 inhibition when sufficient inhibitor concentrations are used, suggesting that the mechanisms underlying chemotherapy resistance and BH3 mimetic sensitivity are not identical.

Analysis of patient cells confirmed this model. We found that the expression of *MYC* and *WT1* oncogenes was downregulated, *TET1* was downregulated, and *CDKN1A* was upregulated, all similar to those observed in the KG1 line. This similarity between in vitro and ex vivo results not only confirms the translational potential of inhibition but also suggests that specific gene and protein signatures (e.g., APAF1, TP53/CDKN1A, and DNMT1/HDAC1 modulation) could serve as biomarkers of treatment response. Since patient samples included both newly diagnosed and refractory cases, we also compared blast cell viability and cell number between these two groups. No statistically significant differences in viability or cell number were observed between newly diagnosed and refractory patients under any treatment condition, indicating that disease status at the time of sample collection did not measurably affect the response to BCL-2/MCL-1 inhibition in this cohort.

The main limitations of this study are the use of ABT-737 instead of venetoclax, which is the clinically approved BCL-2 inhibitor. Nonetheless, ABT-737 is still a commonly used experimental BH3 mimetic that enables the study of BCL-2 family-dependent apoptotic responses in a controlled setting. Although epigenetic changes were noted post-treatment, the direct link between these alterations and the induction of apoptosis has yet to be confirmed. In addition, we cannot exclude the possibility that some of the observed transcriptional changes reflect off-target effects of ABT-737 or S63845 rather than downstream consequences of BCL-2/MCL-1 inhibition itself.

## 5. Conclusions

In conclusion, our study demonstrated that inhibition of BCL-2 and MCL-1 induces coordinated apoptotic and transcriptional responses in AML cells.

## Data Availability

The additional experimental datasets are either included in this manuscript, the Supplemental Information, or are available from the authors upon request.

## Supplementary Materials

The following supporting information can be downloaded at: https://www.mdpi.com/article/10.3390/medicina62071425/s1, Table S1. Primers used for RT-qPCR gene expression analysis; Table S2. Antibodies used for Western Blot analysis; Table S3. AML patient clinical data. Figure S1. IC50 detection. Figure S2. Cell number variation after treatment with a combination of pro-apoptotic agents; Figure S3. Densiometric graphs of proteins Caspase 9, PARP, BAX, BCL-2; Figure S4. Densiometric graphs of proteins SUZ12, EZH2, DNMT1, HDAC1, H4 Hyper Ac, H4K16Ac; Figure S5. BAX/BCL-2 ratio.
