# Supplementary material for: Effects of BCL-2 and MCL-1 Inhibition on Apoptotic and Transcriptional Profiles in Acute Myeloid Leukemia
Source: Medicina (Kaunas). 2026 Jul 22;62(7):1425. doi: 10.3390/medicina62071425 (PMC13414105; doi:10.3390/medicina62071425)
Supplement: Supplementary file 1 [file medicina-62-01425-s001.zip › medicina-4390562-supplementary.pdf]

Supplementary Table S1. Primers used for RT-qPCR gene expression analysis.

Gene name	Sequence of forward and reverse primers (5′ –> 3′)	Product length (bp)
GAPDH	F: AGTCCCTGCCACACTCAG<br>R: TACTTTATTGATGGTACATGACAAGG	123
BCL-2	F: CGGAGGCTGGATGCCTTTG<br>R: TTTGGGGCAGGCATGTTGAC	166
BCL-XL	F:TGCATTGTTCCCATAGAGTTCCA<br>R: CCTGAATGACCACCTAGAGCCTT	79
BAX	F: TGCCTCAGGATGCGTCCACCAA<br>R: CCCCAGTTGAAGTTGCCGTCAG	175
APAF1	F: GGCTGTGGGAAGTCTGTATTAGC<br>R: ACTCTCATCCTGATCCAACCG	165
IDH1	F: TTGGCTGCTTGCATTAAAGGTT<br>R: GTTTGGCCTGAGCTAGTTTGA	115
KAT6A	F: TGTCAGTTTGGGGCATCTCC<br>R: TCTTATGCCGGGAGGAAGGA	73
SIN3A	F: ACAGAAGAGGAGAATTCGGATG<br>R: CGCTCCACGTAGTCTGACC	62
TET1	F: TTCGTCACTGCCAACCTTAG<br>R: ATGCCTCTTTCACTGGGTG	149
HDAC1	F: CAAGCTCCACATCAGTCCTTCC<br>R: TGCGGCAGCATTCTAAGGTT	102
HDAC2	F: AGTCAACGAGGCGGCAAAA<br>R: TGCGGATTCTATGAGGCTTCA	103
GATAD2A	F: GACGGAGACATGAGGGTGAC<br>R: CGTTGTCGGAGAGCACAATCA	202
CDKN1A	F: GGCAGACCAGCATGACAGATT<br>R: GCGGATTAGGGCTTCCTCT	73
TP53	F: TAACAGTTCCTGCATGGGCGGC<br>R: AGGACAGGCACAAACACGCACC	121
WT1	F: GGCATCTGAGACCAGTGAGAA<br>R: GAGAGTCAGACTTGAAAGCAGT	483
MCL-1	F: GTGCCTTTGTGGCTAAACACT<br>R: AGTCCCGTTTTGTCCTTACGA	102
MYC	F: AATGAAAAGGCCCCCAAGGTAGTTATCC<br>R: GTCGTTTCCGCAACAAGTCCTCTTC	112

Supplementary Table S2. Antibodies used for Western Blot analysis.

Antibody	Source/ Clone	Dilution	Manufacturer
Cleaved PARP	Rabbit, clone D64E10	1:1000	Cell Signaling Technology (Danvers, MA, USA)
Cleaved Caspase-9	Rabbit, clone E5Z7N	1:1000	Cell Signaling Technology (Danvers, MA, USA)
Bcl-2	Mouse, clone 124	1:1000	Cell Signaling Technology (Danvers, MA, USA)
Bax	Rabbit, clone D2E11	1:1000	Cell Signaling Technology (Danvers, MA, USA)
DNMT1	Goat, polyclonal	1:500	Santa Cruz Biotechnology (Dallas, TX, USA)
EZH2	Rabbit, polyclonal	1:1500	Thermo Fisher Scientific (Waltham, MA, USA)
SUZ12	Rabbit, clone D39F6	1:1000	Cell Signaling Technology (Danvers, MA, USA)
HDAC1	Mouse, clone 10E2	1:500	Santa Cruz Biotechnology (Dallas, TX, USA)
H4 hyper Ac	Rabbit, polyclonal	1:1000	Millipore (Burlington, MA, USA)
H4K16Ac	Rabbit, polyclonal	1:2000	Millipore (Burlington, MA, USA)
B-Tubulin	Rabbit, polyclonal	1:1500	Abcam (Cambridge, UK)
HRP-conjugated secondary antibody against mouse immunoglobulins	Goat, polyclonal	1:2000	Agilent Dako (Santa Clara, CA, USA)
HRP-conjugated secondary antibody against rabbit immunoglobulins	Goat, polyclonal	1:2000	Agilent Dako (Santa Clara, CA, USA)

Supplementary Table S3. AML patient clinical data.

	Patient 1	Patient 2	Patient 3	Patient 4	Patient 5	Patient 6	Patient 7	Patient 8
**Age**	75	59	75	41	55	50	54	34
**Sex**	Female	Male	Female	Female	Female	Male	Female	Female
**Diagnosis**	AML, not otherwise specified	AML with MDS-related gene mutations	AML with MDS-related gene mutations	AML with NPM1 mutation	AML with MDS-related gene mutations	AML with MDS-related gene mutations	AML with MDS-related cytogenetic changes	AML with NPM1 mutation
**FAB classification**	FAB M0	FAB M1	FAB M2	FAB M5b	FAB M2	FAB M0	FAB M0	FAB M4
**Disease status**	Refractory disease	Newly diagnosed	Newly diagnosed	Newly diagnosed	Newly diagnosed	Refractory disease	Refractory disease	Refractory disease
**Karyotype**	Normal	Normal	Normal	Del(9q)	Normal	trisomy 21	del(5q), monosomy 7, monosomy 22	dup(3p)
**Prior treatment**	1st line: Decitabine (3 Cycles), 2nd line: low-dose Cytarabine+Glasdegib (1 Cycle)	None	None	None	None	Cytarabine + Daunorubicin induction (1 Cycle)	Cytarabine + Daunorubicin induction (1 Cycle)	Cytarabine + Daunorubicin induction (1 Cycle)

**NGS**	No mutations	PTPN11 c.1508G>T (p.Gly503Val), FLT3 c.2028C>A (p.Asn676Lys) and c.1775T>C (p.Val592Ala), RUNX1 c.319C>G (p.Arg107Gly)	SRSF2 NM_003016.4: c.284C>T / p.(Pro95Leu), TET2 NM_001127208.2: c.1835del / p.(Pro612LeufsTer27); c.4894C>T / p.(Gln1632Ter) , c.1772del / p.(Gln591ArgfsTer10), KRAS NM_033360.3: c.99T>A / p.(Asp33Glu), NRAS NM_002524.4: c.37G>T / p.(Gly13Cys)	DNMT3A c.2644C>T (p.Arg882Cys), NPM1 c.860_863dupTCTG (p.Trp288CysfsTer12), FLT3 c.2503G>T (p.Asp835Tyr), FLT3-ITD	IDH2, ASXL1, RUNX1	NRAS c.182A>G (p.Gln61Arg), ASXL1 c.1934dupG (p.Gly646TrpfsTer12), RUNX1 c.319C>T (p.Arg107Cys	No mutations	DNMT3Ac.2645G>A (p.Arg882His), NPM1 c.863_864insCTTG (p.Trp288CysfsTer12), ASXL1 c.2110G>T (p.Gly704Trp)

**Treatment**	low-dose Cytarabine+Glasdegib (1 cycle)	Cytarabine + Daunorubicin induction (1 Cycle), followed by allogeneic stem cell transplantation	Venetoclax + Decitabine (34 cycles)	Cytarabine + Daunorubicin+ Midostaurin induction (1 Cycle), High dose-Cytarabine + Midostaurin consolidation (3 cycles) and Midostaurin maintenance (12 months)	Cytarabine + Daunorubicin induction (1 Cycle), High-dose Cytarabine + Daunorubicin consolidation (1 Cycle), followed by allogeneic stem cell transplantation	Actinomycin D + low-dose cytarabine + venetoclax + trametinib (1 cycle), followed by allogeneic stem cell transplantation	Actinomycin D + low-dose cytarabine + venetoclax + decitabine (2 cycles)	Actinomycin D + low-dose cytarabine + venetoclax (1 cycle)
**Response/ Outcome**	No response/ Died of progressive after 2.5 months	Complete remission/ Alive and in remission for 57 months	Complete remission / Alive and in remission for 57 months	Complete remission/ Alive and in remission for 57 months	Complete remission/ Alive and in remission for 58 months	Complete remission/ Relapse at 2months post-alloSCT, died of progressive disease after 6 months	No response/ Died after 15 months of progressive disease	Morphological leukemia-free state/ Death in aplasia after 1 month

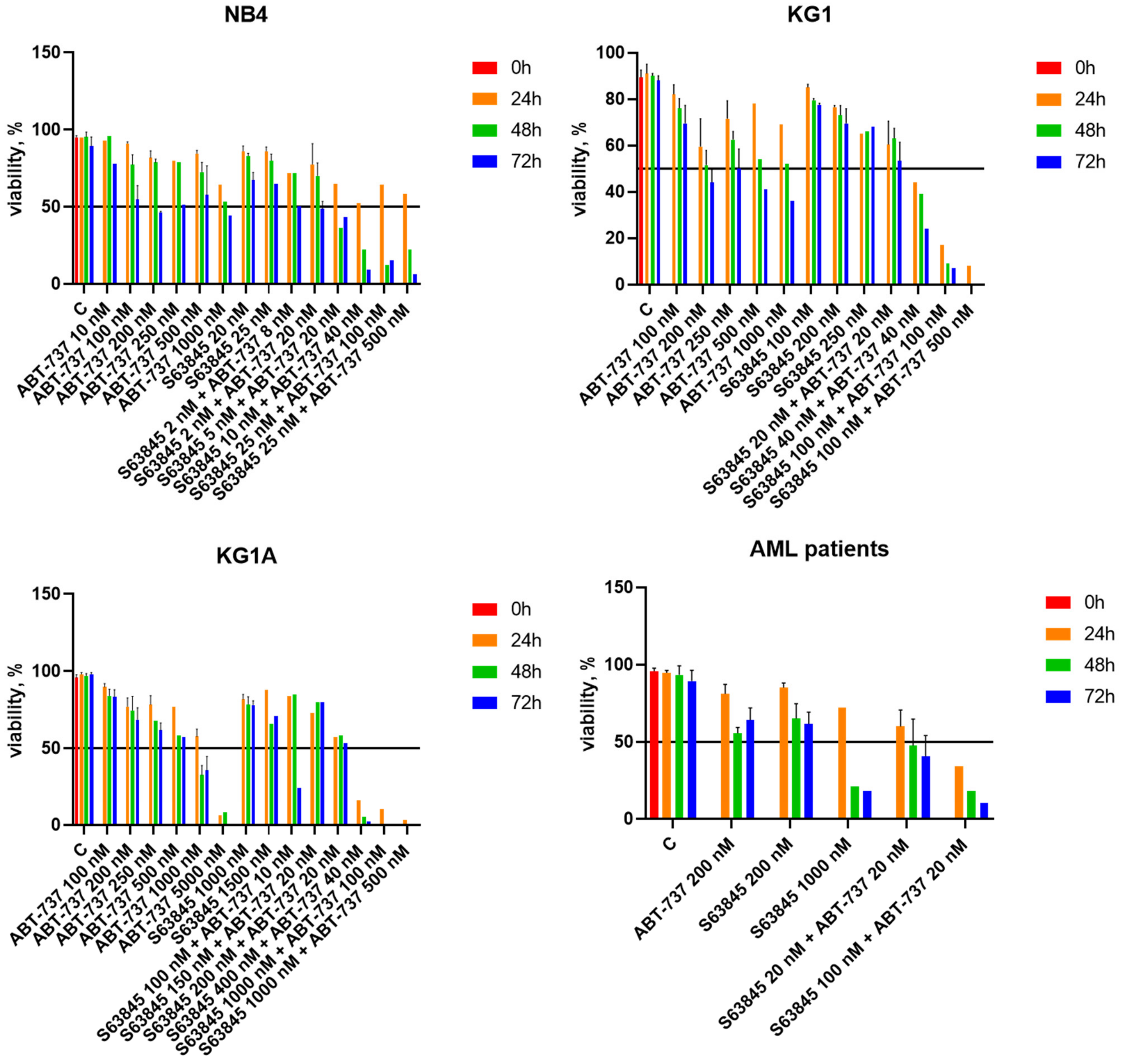

Supplementary Figure S1. IC50 detection graph. Cell viability variation after treatment with a combination of pro-apoptotic agents. Control/C—untreated cells.

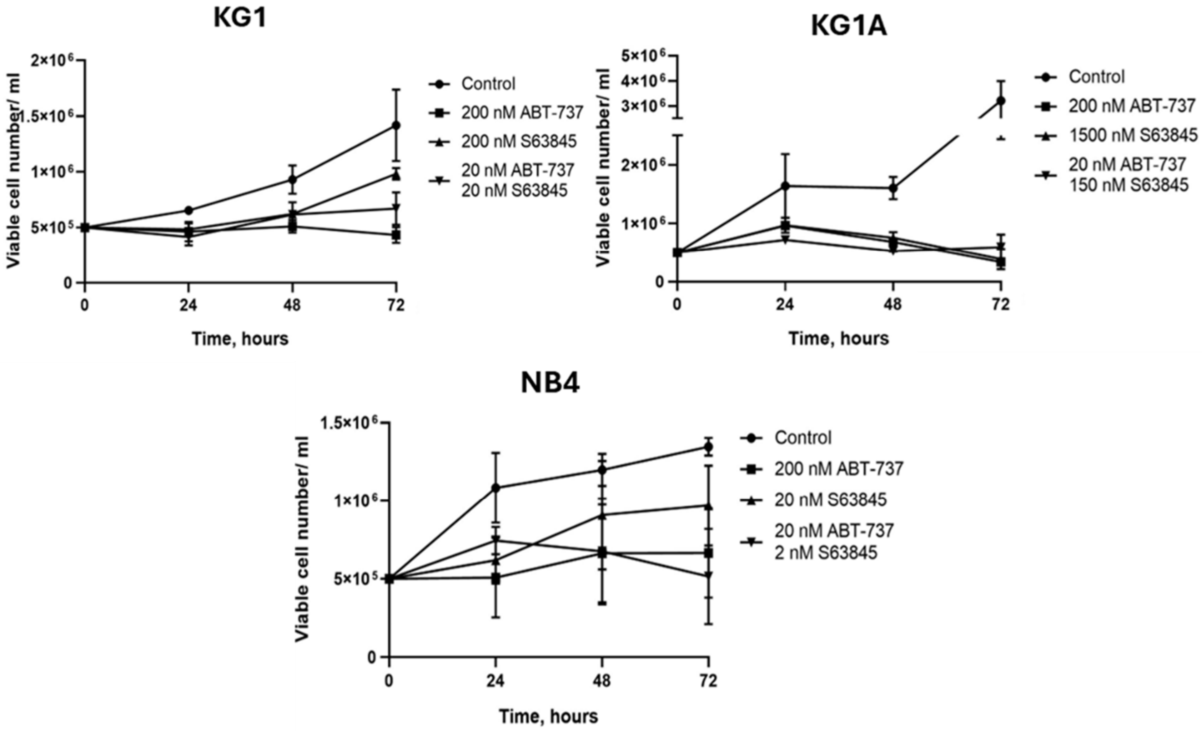

Supplementary Figure S2. Cell number variation after treatment with a combination of pro-apoptotic agents. Control/C—untreated cells. Results are mean ± S.D. (n = 3); statistics calculated by Student's t-test.

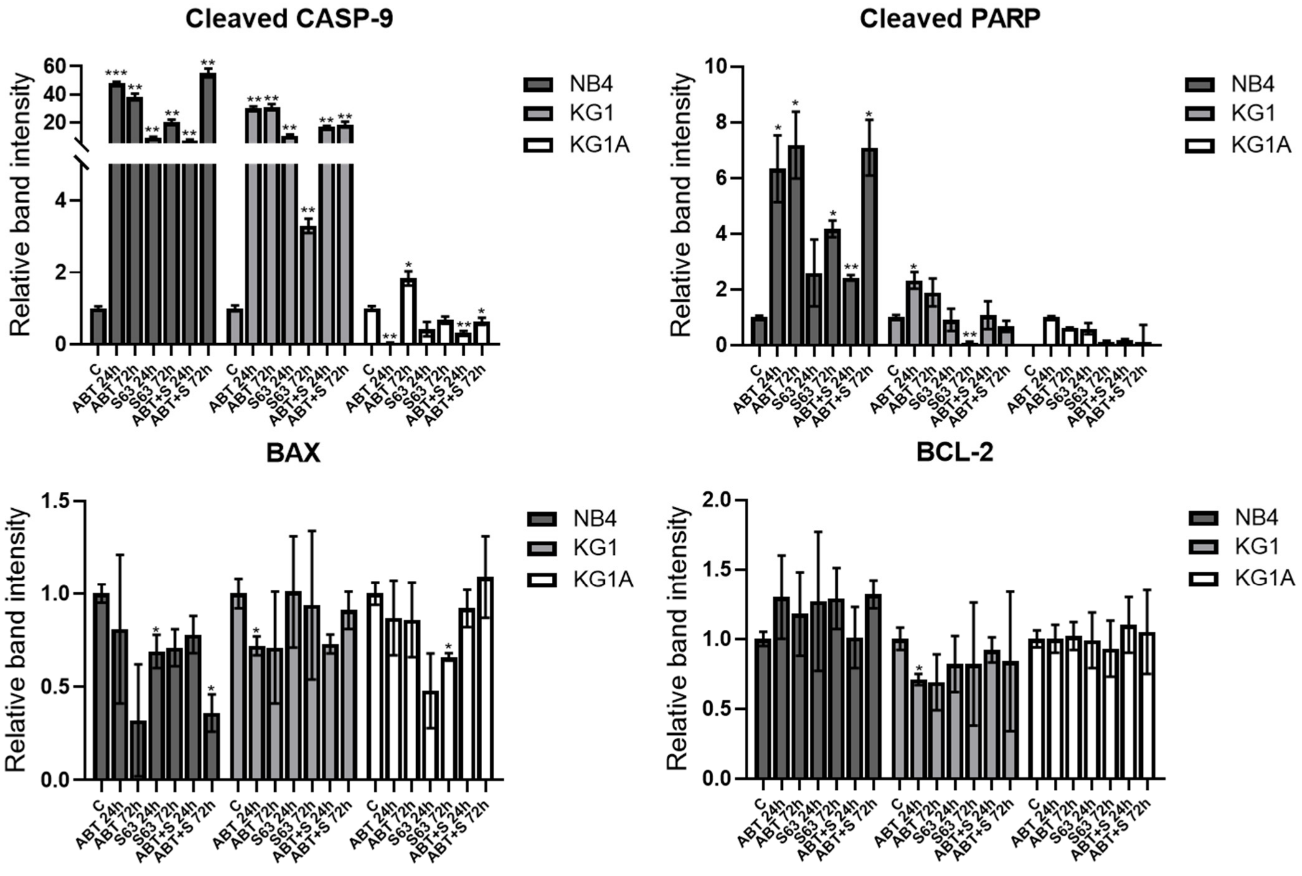

Supplementary Figure S3. Densiometric graphs of proteins Caspase 9, PARP, BAX, BCL-2 levels in NB4, KG1 and KG1A cell lines after treatment with pro-apoptotic agents. ABT—ABT-737; S63—S63845, ABT+S63—combination of agents; C—untreated cells (n=3). NB4—200nM ABT-737, 20nM S63845 or 20nM ABT-737 + 2nM 132 S63845. KG1—200nM ABT-737, 200nM S63845 or 20nM ABT-737 + 20nM S63845. KG1A—200nM ABT-737, 1500nM S63845 or 20nM ABT-737 + 150nM S63845. Statistical significance was determined using one-way ANOVA, $p \leq 0.05$ (*), $p \leq 0.01$ (**), $p \leq 0.001$ (***).

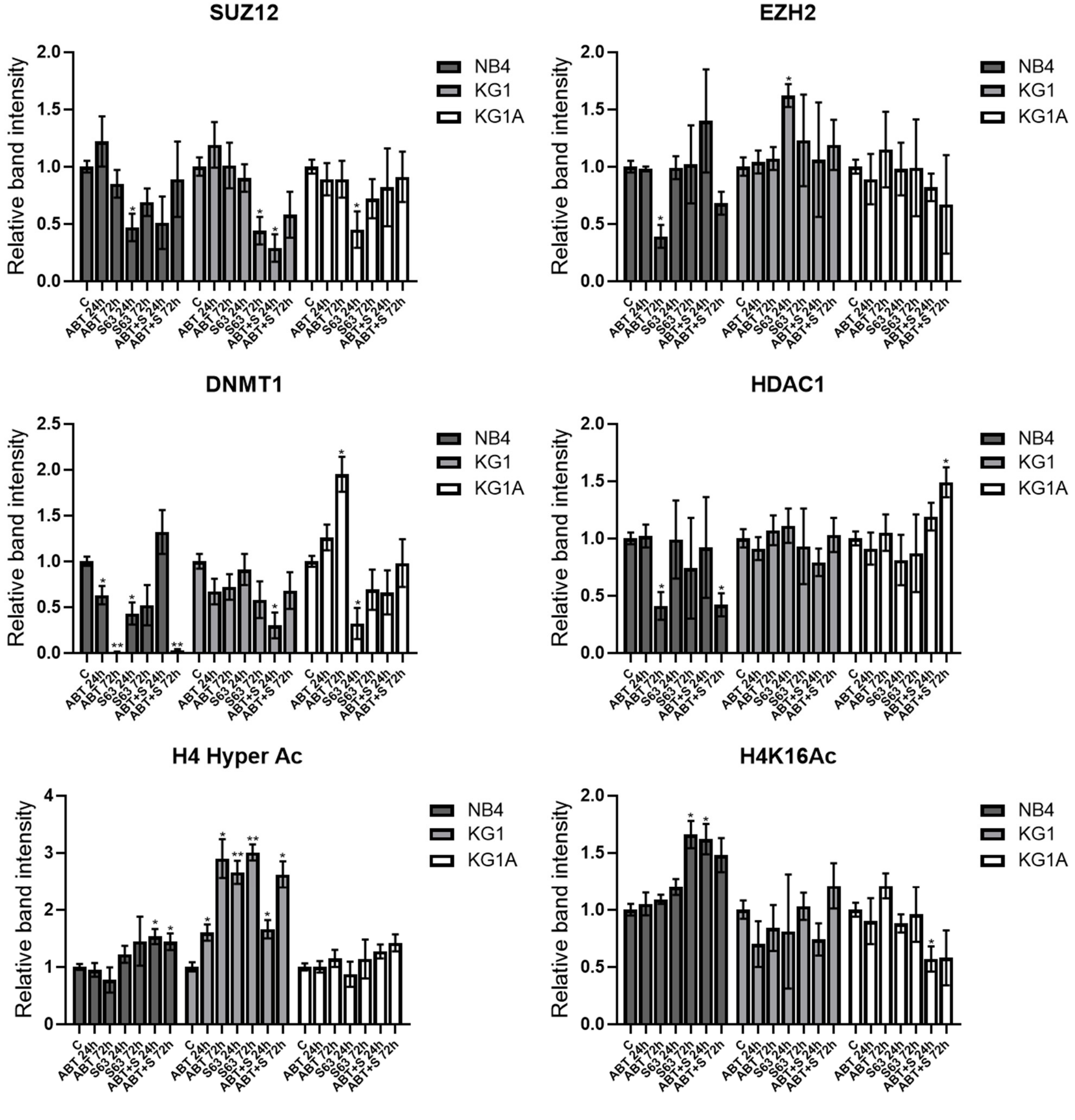

Supplementary Figure S4. Densiometric graphs of proteins SUZ12, EZH2, DNMT1, HDAC1, H4 Hyper Ac, H4K16Ac levels in NB4, KG1 and KG1A cell lines after treatment with pro-apoptotic agents. ABT—ABT-737; S63 – S63845, ABT+S63—combination of agents; C—untreated cells (n=3). NB4—200nM ABT-737, 20nM S63845 or 20nM ABT-737 + 2nM 132 S63845. KG1—200nM ABT-737, 200nM S63845 or 20nM ABT-737 + 20nM S63845. KG1A—200nM ABT-737, 1500nM S63845 or 20nM ABT-737 + 150nM S63845. Statistical significance was determined using one-way ANOVA, p ≤ 0.05 (*), p ≤ 0.01 (**), p ≤ 0.001 (***).

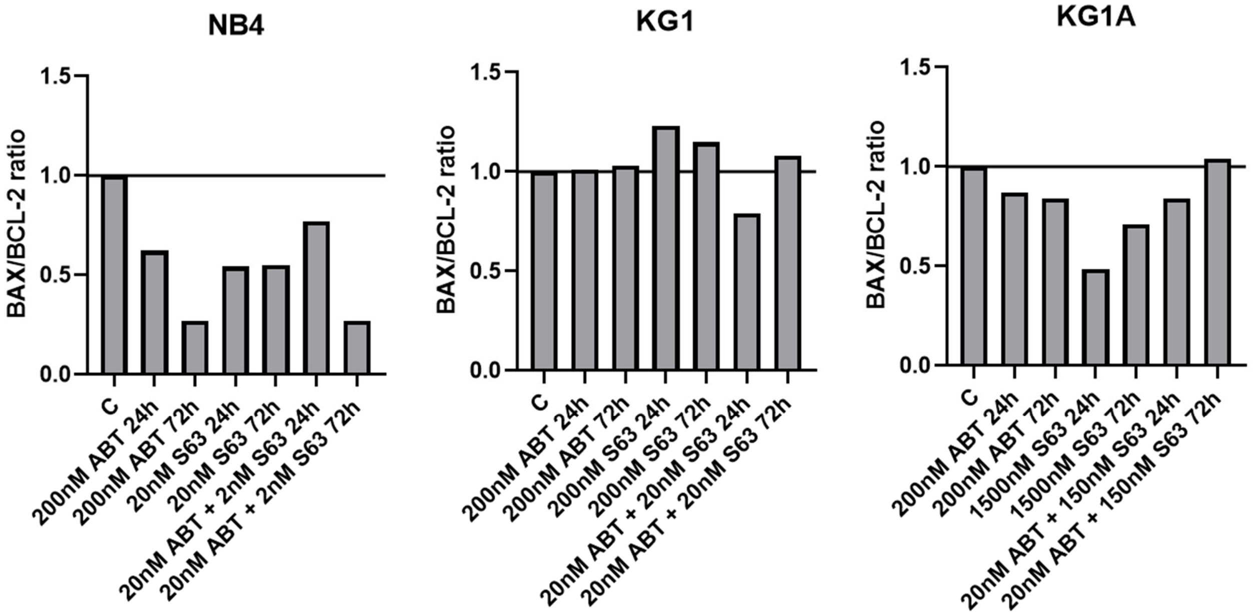

Supplementary Figure S5. BAX/BCL-2 ratio reflects the balance between pro-apoptotic and anti-apoptotic signalling. High-BAX/BCL-2-ratio cells are more prone to death, and low BAX/BCL-2 ratio cells are more resistant to apoptosis.
